# Deep Eutectic Solvents (DESs) as Alternative Sustainable Media for the Extraction and Characterization of Bioactive Compounds from Winemaking Industry Wastes

**DOI:** 10.3390/molecules30081855

**Published:** 2025-04-21

**Authors:** Vincenzo Roselli, Rosalba Leuci, Gianluca Pugliese, Alexia Barbarossa, Antonio Laghezza, Marco Paparella, Alessia Carocci, Vincenzo Tufarelli, Lucia Gambacorta, Luca Piemontese

**Affiliations:** 1 Department of Pharmacy—Pharmaceutical Sciences, University of Bari Aldo Moro, Palazzo “V. Tortorella”, Via E. Orabona 4, 70126 Bari, Italy; 2 Institute of Science of Food Production—ISPA, Research National Council—CNR, Via Amendola, 122/O, 70126 Bari, Italy; 3 Department of Precision and Regenerative Medicine and Jonian Area (DiMePRe-J), Section of Veterinary Science and Animal Production, University of Bari Aldo Moro, 70010 Valenzano, Italy

**Keywords:** winemaking industry wastes, deep eutectic solvents (DESs), green chemistry, antioxidants, antimicrobials, green extraction methods

## Abstract

The increasing pollution and wastage of food and byproducts from agro-industrial production is an increasingly worrying issue. Grape is one of the most diffused fruit crops cultivated, and grape pomace is the main solid byproduct obtained in the winemaking process; interestingly, it is rich in health-beneficial bioactive molecules. In order to recover these molecules, in this work, a green method has been developed, considering two grape pomaces from different cultivars, namely, Petit Verdot and Cabernet Sauvignon. The extraction procedure, as the first step of this process, was carried out with seven selected deep eutectic solvents (DESs). Then, analysis using HPLC-DAD allowed the detection and quantification of eight out of fifteen different phenolic compounds under examination in the extracts produced, including three quercetin glucosides. The evaluation of antioxidant activity, through the DPPH photometric assay, led to the selection of choline chloride/urea 1:2 + 40% water DES extracts as the extracts with the most promising results. Moreover, significant antibacterial activity was also achieved, in particular, for the betaine/lactic acid 1:4 + 40% water DES extract. Further studies will employ this method for numerous cultivars of grape pomaces with the ambitious aim of the production of polyphenol-enriched food and feed supplements.

## 1. Introduction

By the year 2050, the constantly growing global population is predicted to reach a number of up to 11 billion people on Earth [1]. This worrying statistic is associated with large-scale agricultural production with consequently increased emissions, the depletion of water resources and arable lands, and wastage of around 1.3 billion tons of food [1]. About 40–50% of the total amount of wasted food is accounted for by vegetables, fruits, tubers and roots [2,3,4], and a large quantity of inedible parts—consisting mostly of leaves, peels, seeds, press cakes, pits and pulps—are generated by processing fresh fruit and vegetables [2,3,4]. Many reports have documented the high content of health-beneficial bioactive compounds in all these secondary raw materials [2,3,4,5]. The wide spectrum of interesting phytochemicals that can be extracted includes phenolic compounds, which can be further divided into phenolic acids, flavonoids, tannins, lignins, coumarins, and stilbenes [6]. Their importance is certainly due to their antioxidant activity, which guarantees protective effects on the human body, thus contributing, as reducing agents, to the prevention of some chronic oxidative stress-linked diseases such as diabetes, cancer, and cardiovascular and neurodegenerative disorders [6].

Grape is one of the most diffused fruit crops, and more than half (57%) of these crops are used for winemaking [7,8]. Worldwide wine production reached 260 million hectoliters in 2022 [9] (OIV 2022), and, problematically, the wine industry produces high quantities of discarded residues. In particular, 10 g of grapes generates around 120 kg of pomace, the main solid residue [8,10]. This can be a serious issue, considering that grape pomace (GP) contributes to water and groundwater contamination, pollution, and oxygen consumption, slowing down the biodegradation process in landfills because of its antibacterial substances and lower pH [11]. Given these facts, it is imperative to reuse this waste product and to apply its great unused potential. GP is formed by seeds, pulp, skin, and stalks, and it could find use as a precious secondary raw material rich in fibers and in the aforementioned phenolic compounds, since around 70% of them are retained in GP after the winemaking process [11,12].

Nowadays, traditional extraction procedures, such as Soxhlet extraction and maceration, are gradually being replaced by more sustainable and more functional ones [13,14]. It has been demonstrated that innovative extraction processes, such as microwave-assisted extraction (MAE), ultrasonic-assisted extraction (UAE), pulsed electric field-assisted extraction (PEF) and pressurized liquid extraction (PLE), reduce energy consumption along with working time [5]. The extracting solvent must also be chosen carefully to maximize process sustainability. Compared to traditional organic solvents, Deep Eutectic Solvents (DESs) and their sub-category of NAtural Deep Eutectic Solvents (NADESs) are considered a much more advantageous category of extraction media due to their low toxicity, low volatility, and non-flammability, which make their use very safe [5,15]. Moreover, their thermal and chemical stability contribute to their great versatility as well [15]. DESs are made of two or more nontoxic constituents, of which one is a hydrogen-bonding acceptor (HBA) and the other one a hydrogen-bonding donor (HBD), so that the reciprocal hydrogen bond-mediated interaction leads to a consistent reduction in the melting point compared to the starting compounds [16]. NADESs are a type of DESs composed entirely of natural molecules contained in living cells, like sugars, amino acids, and organic acids [16,17].

This research aims to evaluate the ability of seven different DESs to extract bioactive compounds from two different pomaces from red grape cultivars after winemaking. The extracts were characterized from a chemical point of view, through the development of an HPLC-DAD method capable of identifying up to fifteen different phenolic substances. They were also evaluated, when possible, for their antioxidant capacity through a screening 2,2-Diphenyl-1-picrylhydrazyl (DPPH) assay. The samples considered most interesting were then tested for their antimicrobial activity. All determinations were carried out in parallel with the plain DESs and with two types of hydroalcoholic extracts commonly used for the extraction of these matrices. Particularly attractive was the use of NADESs, edible solvents with intrinsic biological properties, which are potentially usable for food and feed supplementation, with evident economic benefits for industries [2,4].

## 2. Results and Discussion

The chemical composition of the two grape pomaces, Cabernet Sauvignon and Petit Verdot, is presented in Table 1. Overall, the two matrices exhibited a comparable chemical–nutritional profile. Moisture and crude protein contents were comparable between the two matrices. Regarding fiber content, both pomaces exhibited high values, characteristic of grape residues. Crude lipid content followed a similar pattern, being slightly higher in Cabernet Sauvignon than in Petit Verdot (5.23 vs. 4.57% DM). Ash content, indicative of the total mineral fraction, was slightly higher in Petit Verdot compared to Cabernet Sauvignon (8.80 vs. 7.82% DM). These results indicated that the two grape pomaces have a comparable bromatological composition, suggesting that varietal differences exert a limited influence on the overall nutritional profile. Such observations are also consistent with previous findings, which reported similar proximate compositions among different grape pomace varieties [18,19].

**Figure 1:**
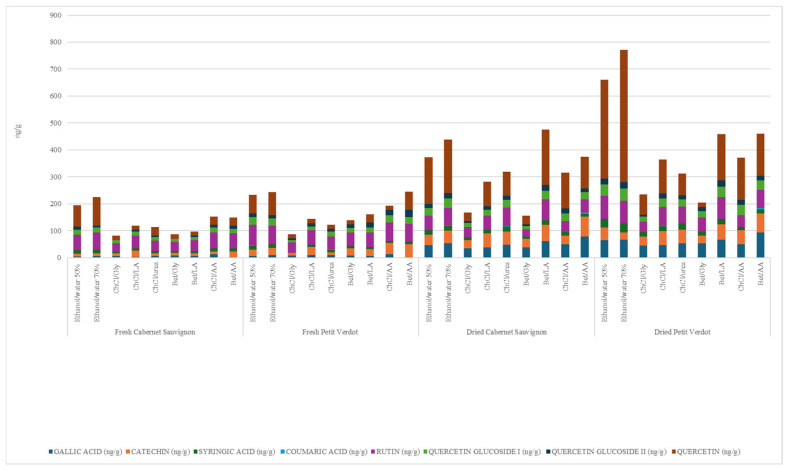
Sum of levels of the eight polyphenols in fresh and dried Cabernet Sauvignon and in fresh and dried Petit Verdot grape pomace according HPLC-DAD analysis; all the DESs included +40% water.

According to HPLC-DAD characterization (Table 2, Figure 1), six out of thirteen phenolic compounds’ standards were present in the considered sample (and eight bioactive substances out of fifteen, including the two additional quercetin glucosides).

Quercetin was the most abundant polyphenol, and it was present in all samples with a maximum quantity of 492.5 ng/g in dried Petit Verdot ethanol 70% extract, while coumaric acid was the least abundant one, found only in Bet/AA + 40% water NADES’s extracts from both dried GPs at a level of 5.2 ng/g. 

The analysis of the UV spectra suggested the presence of three different quercetin glucosides as well, with rutin (as identified with the specific standard fortification) being the most abundant of the three in all samples, reaching a level up to 84.6 ng/g when using ethanol/water 50% on dried Petit Verdot GP. The analysis confirmed that, considering the total amount of phenolic compounds detected in each sample, the two reference amounts, ethanol/water 50% and ethanol/water 70%, were the best extracting solvents for phenolic compounds recovery, although NADES Bet/LA + 40% water reached higher yields (475.3 ng/g in total of polyphenols) in dried Cabernet Sauvignon GP, therefore being the best solvent in that case (Figure 1). Moreover, between these two reference extracting solvents, ethanol 70% was more effective in each matrix (fresh and dried Cabernet Sauvignon and fresh and dried Petit Verdot) than ethanol 50%. 

Among all DESs/NADESs experimented on, AA-based NADESs (ChCl/AA + 40% water, Bet/AA + 40% water) produced interesting results, being among the best able to earn the highest levels in all extractions. Bet/AA + 40% water NADESs obtained higher yields than ChCl/AA + 40% water NADESs for both dried GPs and fresh Petit Verdot GP, while in fresh Cabernet Sauvignon, they displayed similar results (149.8 ng/g and 152.5 ng/g, respectively), considering, however, a total absence of gallic acid in the Bet/AA + 40% water NADES extract, which was compensated by a higher quantity of catechin present (23.4 ng/g compared to <LOQ in the ChCl/AA + 40% water NADES extract). 

For dried Petit Verdot GP, Bet/AA + 40% water and Bet/LA + 40% water NADESs showed similar yields (457.8 ng/g for Bet/AA and 459.6 ng/g for Bet/LA) with higher levels of syringic acid, quercetin, and quercetin glucosides for Bet/LA + 40% water NADES and higher amounts of catechin and gallic acid for Bet/AA + 40% water NADES. Glycerol-based DESs (ChCl/Gly + 40% water, Bet/Gly + 40% water) were among the least effective for polyphenol extraction. In particular, they had the worst extracting capacity in three out of all four cases (dried Petit Verdot and fresh and dried Cabernet Sauvignon). The second worst extracting solvent was found to be ChCl/urea + 40% water, especially in fresh Petit Verdot GP extraction.

The recent literature has already reported various DESs for the extraction of polyphenols from dried grape pomace derived from other European cultivars. In particular, the extraction of polyphenols can be enhanced by using ultrasonic pre-treatment and an appropriate content of water and polarity of DESs, as determined by the choice of suitable HBA to combine with glycerol as an HBD [20]. In another case, the combination of betaine or choline chloride with citric acid or urea gave a higher total polyphenol content than a hydroalcoholic mixture of ethanol/water 50% [21]. NADESs composed of betaine or proline with lactic acid are capable of extracting polyphenols, with a higher concentration of acids such as gallic acids and syringic acid [22]. These studies confirmed that betaine-based DESs are promising extraction media [21,22].

**Figure 2:**
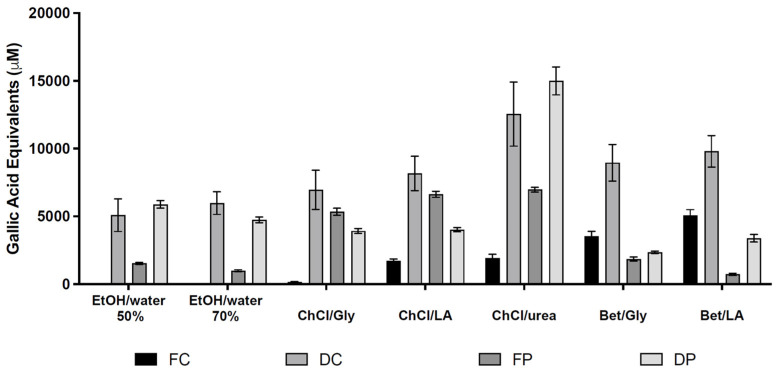
Antioxidant activity calculated, for each extract, as gallic acid equivalents (GAEs) through DPPH photometric assay. FC: fresh Cabernet Sauvignon; DC: dry Cabernet Sauvignon; FP: fresh Petit Verdot; DP: dry Petit Verdot.

Contrary to what was observed through HPLC-DAD analysis, the DPPH photometric assay (Table 3, Figure 2) showed that aqueous ethanol solutions have much lower radical scavenging activity compared to DESs/NADESs systems.

In fact, even though for the fresh Cabernet Sauvignon hydroalcoholic extracts the determination was not possible (as the extracts were unavailable), the corresponding fresh Petit Verdot solutions showed low antioxidant profiles (1530 and 990 μM GAE). The results for dried GP ethanolic extracts were similar (between 4740 μM GAE and ethanol 70% for Petit Verdot and 5980 μM GAE and ethanol 70% for Cabernet Sauvignon). These data were among the lowest detected values in dried Cabernet Sauvignon-derived samples. This trend was retained when observing the data obtained from fresh Petit Verdot GP: only NADES Bet/LA + 40% water had lower antioxidant activity (720 μM GAE) than the hydroethanolic extracts. On the other hand, ethanol 50% and ethanol 70% were found to have, respectively, the second (5880 μM GAE) and the third best results (4740 μM GAE) in terms of antioxidant activity, though their activities were significantly lower than that of the ChCl/urea + 40% water NADES extract of dried Petit Verdot GP (15,000 μM GAE).

Among all the DESs, ChCl/urea + 40% water has been confirmed to be the best choice for preserving the highest antioxidant activity in almost all experiments. Only in the case of fresh Cabernet Sauvignon GP, Bet/Gly + 40% water and Bet/LA + 40% water DESs’ extracts were able to do better (3530 and 5060 μM GAE, respectively, compared to 1910 μM GAE for ChCl/urea + 40% water). On the other hand, for dried Cabernet Sauvignon GP, Bet/LA + 40% water and Bet/Gly + 40% water DES extracts were also very effective, showing the second (9800 μM GAE) and the third (8960 μM GAE) best results, respectively. Instead, ChCl/Gly + 40% water DES extracts displayed the lowest antioxidant activity for both dried (6960 μM GAE, but better than hydroalcoholic samples) and fresh (160 μM GAE) Cabernet Sauvignon GPs.

With respect to fresh and dried Petit Verdot extracts, ChCl/LA + 40% water and ChCl/Gly + 40% water DESs were confirmed to be able to retain high antioxidant activity (6630 and 5340 μM GAE for fresh GP and 4020 and 3920 μM GAE for dried GP, respectively). Furthermore, the Bet/Gly + 40% water DES extract showed one of the worst results for both matrices (2350 μM GAE for fresh and 1850 μM GAE for dried).

The excellent antioxidant activity of DES extracts compared to hydroalcoholic or alcoholic extracts has already been reported in the literature, and our studies seem to confirm it, although it is difficult to compare our results with previous ones because of differences in the performed assays and/or data expression [21,22,23].

Unfortunately, the evaluation of antioxidant activity for AA-based NADESs was not possible because, even at very low concentrations, AA displayed very high activity itself in the experimental conditions. Consequently, the calculation of their EC_50_ values was not allowed. For other samples, the influence of the media on the antioxidant activity was evaluated as well; ChCl-based solvents were completely inactive, except for ChCl:LA + 40% water (15 μM GAE), while Bet-based solvents had a noticeable (143 and 192 μM GAE for LA and Gly, respectively) but not overt effect. In any case, the use of media intrinsically capable of having a certain biological activity (i.e., antioxidant) is a plus for possible industrial applications of the whole extract as an additive or supplement.

The best results were obtained when using extracts from dried GPs. This seems obvious, considering that, with weight being the same, higher contents of bioactive substances were to be expected from dried samples. However, this was not certain, considering the possibility of losing part of these substances during the drying process of the matrices, which is very important for their conservation. Red grape pomace is in fact often attacked by mold, both pre-harvest and post-harvest, if not properly stored [24]. Our results show that drying is a strategy that could prolong its shelf life while preserving the antioxidant capacity of its extracts.

Finally, *in vitro* antibacterial assays were also performed on the most promising extracts from dried GPs, selected considering their phenolic and antioxidant profiles. Recent studies have demonstrated that grape pomace-derived polyphenols exhibit strong antibacterial effects against both Gram-positive and Gram-negative bacteria [25]. This effect was investigated by means of the microdilution method following the Clinical and Laboratory Standards Institute (CSLI) methodology against six bacterial strains belonging to the American Type Culture Collection: *Enterococcus faecalis* 29212 (Gram-positive), *Staphylococcus aureus* 29213 (Gram-positive), 43300 (Gram-positive), *Escherichia coli* 25922 (Gram-negative), *Klebsiella pneumoniae* 13883 (Gram-negative), and *Pseudomonas aeruginosa* 27853 (Gram-negative).

Considering that previous studies have documented the inhibitory effects of DESs towards different microorganisms [26], we analyzed, in parallel, the activity of pure media (ChCl/AA + 40% water, Bet/LA + 40% water, ChCl/Urea + 40% water, and 70% EtOH) in the same dilution range applied for the extracts.

Levofloxacin, a well-known broad-spectrum fluoroquinolone antibiotic, was used as a reference due to its established efficacy against both Gram-positive and Gram-negative bacteria. Its inclusion ensures the reliability and validity of the test by providing a reference for assessing the antimicrobial activity of the extracts.

Table 4 and Table 5 report the obtained data expressed as a minimum inhibitory concentration (MIC) % *v*/*v*—defined as the lowest concentration of samples where no growth is observed—and minimum bactericidal concentration (MBC) % *v*/*v*—defined as the lowest concentration of the compound at which no visible bacterial growth was observed on the agar plates, indicating that ≥99.9% of the initial bacterial population had been killed—for DESs systems themselves and 70% EtOH and grape pomace extracts. The selected extracts were obtained from both dried Cabernet Sauvignon (140) and Petit Verdot (119, 122 and 152) GPs.

These results indicate that among the tested DESs, Bet/LA + 40% water possesses the highest intrinsic activity against all tested bacterial strains.

With regard to the extracts, the results reveal some differences in their antimicrobial efficacy depending on the extract and bacterial strain tested. Extract 140, obtained from dried Cabernet Sauvignon GP using Bet/LA + 40% water as an extraction solvent, exhibited the most interesting antibacterial activity, with MIC values ranging from 0.1 to 0.4% *v*/*v* and MBC values from 0.2 to 0.8% *v*/*v*. This suggests a strong inhibitory and bactericidal effect against all tested strains of both Gram-positive and Gram-negative bacteria. Notably, this extract shows a remarkable improvement in antibacterial activity compared to the bare NADES, which exhibited MIC values between 0.4 and 0.8% *v*/*v* and MBC values between 0.8 and 1.6% *v*/*v*, demonstrating the antimicrobial activity of the grape pomace extract particularly when extracted in Bet/LA + 40% water. The most pronounced activity was observed against *S. aureus* strains, including *S. aureus* ATCC 43300, a methicillin-resistant *S. aureus* (MRSA) strain. This is particularly significant given the clinical relevance of MRSA as a major cause of hospital- and community-acquired infections, often associated with multidrug resistance. Moreover, the observed activity against *P. aeruginosa* ATCC 27853 is also noteworthy, since it is comparable to the effect observed against Gram-positive strains. This is significant because *P. aeruginosa* is an opportunistic pathogen with a high level of intrinsic antibiotic resistance, due to its low outer membrane permeability and active efflux mechanisms.

Notable antibacterial activity was also displayed by Extract 122, obtained from dried Petit Verdot GP using ChCl/AA + 40% water as a solvent. Indeed, MIC values ranged from 0.4 to 1.6% *v*/*v,* and MBC values ranged from 0.8 to 3.1% *v*/*v*. This extract demonstrated good efficacy against all tested strains, with a slight preference for Gram-positive bacteria. The solvent alone showed MIC values ranging from 1.6 to 6.3% *v*/*v* and MBC values between 3.1 and 12.5% *v*/*v*. Again, the best results were observed against *S. aureus*, with an MIC of 0.4% *v*/*v*. Notably, this effect was comparable in both the methicillin-sensitive (*S. aureus* ATCC 29213) and methicillin-resistant (*S. aureus* ATCC 43300, MRSA) strains.

Extracts 119 and 152, both obtained from dried Petit Verdot GP using 70% EtOH and ChCl/Urea + 40% water as solvents, demonstrated moderate antibacterial activity and a similar trend in results, with MIC values between 0.4 and 3.1% *v*/*v* and MBC values between 0.8 and 6.3% *v*/*v*. These extracts were more effective against Gram-positive strains, such as *S. aureus* ATCC 29213 and *S. aureus* ATCC 43300, compared to Gram-negative strains. When compared to their respective solvents alone, both extracts exhibited significantly lower MIC and MBC values. The MIC values of the ethanol control ranged from 12.5 to 25% *v*/*v*, while ChCl/Urea + 40% water had MIC values between 6.25 and 12.5% *v*/*v*.

These findings indicate that extract 140, obtained with Bet/LA 1:4 + 40% water, possesses the strongest antibacterial activity among the analyzed grape pomace extracts, suggesting its potential application as a natural antimicrobial agent. Furthermore, the comparison with solvent controls highlights the significant role of grape pomace bioactive compounds in enhancing antimicrobial efficacy, making them promising candidates for further investigation in antibacterial applications.

The interesting results obtained in this study corroborate previous research, highlighting the antimicrobial potential of grape pomace extracts and the impact of NADES on bioactive compound extraction. In particular, Bet/LA + 40% water as the extraction solvent has been shown to enhance polyphenol solubility and bioavailability, potentially explaining the superior antimicrobial activity observed in extract 140 [22]. Furthermore, these results confirm previous findings that DESs themselves possess antimicrobial properties, as seen in the MIC and MBC values of the solvent controls [26,27]. It is notable that, when combined with grape pomace extracts, these values were significantly reduced compared to the DES alone. Hence, it is reasonable to assume that these green solvents could contribute to the overall antibacterial activity of the bioactive compounds present in the extracts, as also hypothesized in previous studies [26,27,28,29]. Additionally, the previous literature has indicated that ethanol-based extracts of grape pomace show only moderate antibacterial activity, particularly against *S. aureus* and *E. coli* [28].

## 3. Materials and Methods

### 3.1. Sampling and Instrumentation

The Cabernet Sauvignon (CS) and Petit Verdot (PV) grape pomaces (GPs) were collected during the 2023 season to develop a method to fully characterize the wine industry’s main solid waste. All the chemicals used were of analytical grade, and they were purchased from common suppliers and used without further purification.

Chemical characterization of the GPs was carried out by employing an Ankom 220 Fiber Analizer (ANKOM Technology, Macedon, NY, USA) for crude fiber (CF) determination, fully automatic Bherotest Steam distillation apparatus S5 (Behr Labor-Technik, Düsseldorf, Germany) equipped with an external titrator TitroLine^®^ 5000 (SI Analytics, Xylem Inc., Washington, DC, USA) for crude protein (CP) determination, and Soxhlet apparatus (LAUDA, Lauda-Königshofen, Germany) for crude lipids. DES/NADES preparation was performed using a magnetic stirrer and a rotary evaporator (Heidolph Hei-VAP Core (Heidolph, Schwabach, Germany)) equipped with a PC 3001 VARIO select vacuum pump (Vacuubrand, Wertheim, Germany). The extraction procedure was carried out through an orbital shaker (PSU-10i, BIOSAN, Riga, Latvia) and an ALC PK 121R multispeed refrigerated centrifuge (ALC International, Cologno Monzese, Italy).

The HPLC-DAD analyses were performed with the use of an Agilent 1260 Infinity liquid chromatograph (Agilent Technologies Inc., Wilmington, DE, USA) equipped with a binary pump (G1312B), an auto sampler (G1367E) with a 100 μL loop, a spectrophotometric diode array detector (DAD) (G4212B), and software for Microsoft Windows 7 (OpenLAB, CSB, ChemStation Edition). The separation was achieved with a Zorbax SB-C18 column 5 μm (4.6 × 150 mm) (Agilent Technologies, Inc.). The DPPH photometric assay was carried out with a UV–Vis spectrophotometer and Infinite M1000 Pro multiplate reader (Tecan, Cernusco S.N., Italy).

### 3.2. Preparation of Extraction Media

Seven DESs were prepared using choline chloride (ChCl) and betaine hydrochloride (Bet) as HBAs, and glycerol (Gly), lactic acid (LA), urea, and ascorbic acid (AA) as HBDs. Table 6 briefly reports the DESs’ composition. In all DESs, 40% water content was used because a lower percentage does not significantly affect polar molecules’ recovery, and a lower viscosity can be achieved [30].

Ascorbic acid-based NADESs were prepared using the procedure described by Liu W. et al. [31] with modifications. Each of the two components was dissolved in water at room temperature for 20–30 min until a solution was obtained. Then, the two solutions were mixed, and the excess of water was eliminated using rotary evaporator apparatus (40 °C, 50 mbar, 60–120 min). Bet/Gly + 40% water and Bet/LA + 40% water DESs were prepared by modifying the procedure applied by Liu Y. et al. [32]. The optimized method consists of the same steps followed for AA-based NADESs but with changing rotary evaporator operating conditions (55 °C, 80 mbar, 60–120 min).

The other ChCl-based DESs (ChCl/Gly + 40% water, ChCl/LA + 40% water, ChCl/urea + 40% water) [21,33,34] were made by mixing and stirring (80–90 °C, 30–60 min) the corresponding two components until the formation of a homogenous transparent solvent. Finally, the calculated quantity of water was added (Table 6).

Two types of aqueous ethanol solutions were prepared as classical solvents in the extractions: ethanol/water 50% (*wt*/*wt*) [35,36,37] and ethanol/water 70% (*wt*/*wt*) [38,39,40].

### 3.3. Chemical Analyses

The content of dry matter (DM, method 945.15; AOAC [41]) was determined by a moisture analyser at 35 °C for 72 h (mild conditions were chosen in order to simulate field conditions). The Kjeldahl method (990.03) [42] was used to determine CP, which was calculated as nitrogen × 6.25 [42] on a dry matter basis. The method of Van Soest et al. [43] was employed to determine CF content. Total ash determination was performed by weighting 3 g of representative samples and placing them in a muffle furnace for 4 h at 550 °C (method 967.05) [44]. Crude lipids (method 922.06) were determined by extracting the fat from the sample using petroleum ether as a solvent [45,46].

### 3.4. Extraction

All the preliminary experiments were conducted on both fresh and dried GPs to verify that dried ones also had good yield and antioxidant activity, which could be important information since the storage of dried GPs is easier and less energy-intensive. In fact, they can be stored at room temperature with a low risk of microbial contamination [47].

Homogenous samples of both fresh and dried GP were weighted (5 g) and mixed with DESs or NADESs at a 1:4 (*wt*/*wt*) ratio. The experiment was performed for 1 h at room temperature under shaking (230 rpm) in closed jars, as similarly reported by Chamorro et al. [48]. In the following step, the solid was excluded from the extract through centrifugation (4500 rpm, 25 °C, 10 min) and decantation before storing the samples in plastic tubes (1.5 mL) at −80 °C for further analyses. All experiments were carried out in triplicate.

### 3.5. Quali-Quantitative Determination of Bioactive Compounds

High-Performance Liquid Chromatography (HPLC) was used to detect and quantify the extracted phenolic compounds, in particular gallic acid, catechin, syringic acid, rutin, quercetin, kaempferol 3-galactoside, apigenin, apigenin 3-glucuronide, resveratrol, ferulic acid, chlorogenic acid, caffeic acid and coumaric acid. The extracts were diluted with water (1 mL of water for 1 mL of extract), centrifuged (14,400 rpm, 30 °C, 2 min), and filtered through 0.45 μm regenerated cellulose filters. The method and the parameters used were based on that of Sergio et al. [49] with some modifications. A volume of 20 μL of each sample was injected at 25 °C in the column. The flow rate was fixed at 0.7 mL/min (25 °C), and the mobile phase consisted in a binary gradient of acidic H_2_O (containing 5% acetic acid) (A) in MeOH (B) (Table 2). The gradient was set as follows: from 2% to 40% MeOH in 35 min; maintained at 40% MeOH for 5 min; brought to 63% MeOH in 15 min; maintained at 63% MeOH for 7 min; and then brought to 100% MeOH in 4 min and maintained at 100% MeOH for 1 min. The MeOH was brought to 2% in 8 min and left to equilibrate for 5 min before the next run. Phenolic compound standard solutions were prepared using methanol as a solvent. Detection was carried out simultaneously at five different wavelengths (260, 280, 325, 360, and 560 nm). The limit of detection (LOD) and quantification (LOQ) values of each phenolic compound were calculated at signal-to-noise ratios of three and six, respectively (Table 7).

### 3.6. Detection of Antioxidant Activity

Antioxidant activity was determined as relative antioxidant activity using the DPPH photometric assay. A previously reported procedure, used by Carocci et al. [50], which was inspired by the one developed by Blois et al. [51], was carried out with some modifications. A volume of 100 μL of extract (1:50, 1:150, 1:450, 1:1350, 1:4050, 1:12,150, 1:36,450, *v*/*v*) diluted with water/methanol (50:50) was mixed with 100 μL of 200 μM DPPH solution in methanol obtained from freshly prepared 10 μM stock. The mixtures were incubated in dark conditions for 30 min at room temperature. The absorbance measurement was performed by setting the UV–Vis spectrophotometer to 640 nm, since at the conventional wavelength (520 nm), the absorption could be affected by the colour of the extracts at the highest concentrations. Results were expressed in μM gallic acid equivalents (GAEs) after abuilding a gallic Acid standard curve (100 μM, 40 μM, 16 μM, 6.4 μM, 2.56 μM, 1.02 μM, 0.41 μM). All analyses were carried out in duplicate.

### 3.7. Determination of Antibacterial Activity

The *in vitro* minimum inhibitory concentrations (MICs, μg/mL) and the minimum bactericidal concentration (MBCs, μg/mL) were determined following the Clinical and Laboratory Standards Institute (CLSI) guidelines by means of the broth microdilution method [52]. A concentration gradient (50 to 0.05% *v*/*v*) was established in the wells using two-fold serial dilutions by using Mueller Hinton broth (MHB, Oxoid, Milan, Italy) as a medium. The following bacterial strains from the American Type Culture Collection (ATCC, Rockville, MD, USA), which were available as freeze-dried discs, were used: *E. faecalis* (ATCC 29212), *S. aureus* (ATCC 29213, ATCC 43300), *E. coli* (ATCC 25922), *K. pneumoniae* (ATCC 70063), and *P. aeruginosa* (ATCC 27853). The turbidity of the bacterial cell suspension was adjusted to match the 0.5 McFarland standard using a spectrophotometer (OD625 nm readings between 0.08 and 0.10). The standardized suspension underwent an additional 100-fold dilution in MHB, resulting in a final concentration of 1–2 × 10^6^ colony-forming units (CFUs) per milliliter. Subsequently, 100 μL aliquots of the final inoculum were introduced into each well. The experiment included two controls: a medium sterility control (comprising only the medium) and positive control (containing the bacterial suspension in the medium). Levofloxacin (Sigma Aldrich, Milan, Italy) served as the standard reference antibiotic for this study. The plates were incubated at 37 °C for 24 h. MIC values were determined as the lowest concentration of the compounds at which no visible bacterial growth was observed [53]. In addition, the antibacterial activity of each deep eutectic solvent (DES), used as the solvent for the tested extracts, was evaluated across the same concentration range as the extracts themselves. To determine the MBC values, bacterial cultures were treated with varying concentrations of the test compounds and incubated under the same conditions as the MIC assay. After incubation, 10 μL from each well showing no visible bacterial growth was plated onto fresh Mueller–Hinton agar and incubated at 37 °C for 24 h. The MBC was defined as the lowest concentration at which no visible bacterial colonies were observed on the agar surface, indicating a ≥99.9% reduction in the initial bacterial population. All experiments were performed three times in duplicate to ensure reproducibility.

## 4. Conclusions

The employment of DESs and NADESs as alternative and green extracting solvents was evaluated for the development of a targeted method for the recovery of phenolic compounds from grape pomace. Cabernet Sauvignon and Petit Verdot grape pomaces were collected during the 2023 vintage, and their chemical composition analysis (dry matter content, crude fiber, crude protein, crude fat, and total ash) was carried out, revealing similar values for both matrices considered. Seven DESs or NADESs, all with a water content of 40% (*w*/*w*), were prepared by employing ChCl and Bet as HBAs, and Gly, LA, urea, and AA as HBDs. On the other hand, aqueous ethanol solutions (ethanol 50% and 70% *w*/*w*) were used for comparison. Despite the higher yields of phenolic compounds detected through HPLC-DAD, in the extracts of aqueous ethanol solutions, DES/NADES extracts generally showed very good recoveries as well. In both fresh GP samples, AA-based NADESs were found to be the best choice, while in dried GPs, Bet/LA 1:4 + 40% water and ChCl/AA 1:2 + 40% water DESs reached the highest extraction yields. Furthermore, the evaluation of antioxidant activity through a DPPH photometric assay revealed that ChCl/Urea 1:2 + 40% water was the most promising solvent in this regard. Finally, the ethanol-extracted pomace (extract 119, obtained from dried Petit Verdot GP) exhibited similar trends, though its efficacy was exceeded by several DES-based extracts. This suggests that, while conventional solvents like ethanol are effective, DESs may be a superior alternative due to their ability to extract a broader range of bioactive compounds while maintaining biocompatibility. Furthermore, extract 140, obtained from dried Cabernet Sauvignon GP using Bet/LA 1:4 + 40% water, showed the strongest antibacterial activity, indicating its potential application as a natural preservative agent.

Future studies will be carried out on a higher number of different grape pomaces by employing this method and the selected extracting solvents, with the aim of using the best products for the formulation of polyphenol-enriched foods and feeds or using the whole extracts as dietary supplements.

## Figures and Tables

**Table 1:** Chemical composition of grape pomaces under study (mean ± standard error).

Grape Pomace	Moisture %	Crude Protein % DM *	Crude Fiber % DM *	Crude Lipids % DM *	Total Ash % DM *
Cabernet Sauvignon	48.85 ± 0.47	6.32 ± 0.15	35.09 ± 0.29	5.23 ± 0.09	7.82 ± 0.18
Petit Verdot	50.33 ± 0.39	5.49 ± 0.13	34.61 ± 0.31	4.57 ± 0.11	8.80 ± 0.16

* DM: dry matter.

**Table 2:** Phenolic compound contents.

GRAPE POMACE	SOLVENT	GALLIC ACID (ng/g)	CATECHIN (ng/g)	SYRINGIC ACID (ng/g)	COUMARIC ACID (ng/g)	RUTIN (ng/g)	QUERCETIN GLUCOSIDE I (ng/g)	QUERCETIN GLUCOSIDE II (ng/g)	QUERCETIN (ng/g)
Fresh Cabernet Sauvignon	Ethanol/water 50%	4.9	<LOQ	12.8	ND	57.3	17.6	12.0	78.7
	Ethanol/water 70%	6.1	<LOQ	14.1	ND	63.2	18.2	6.8	106.8
	ChCl/Gly	5.6	<LOQ	6.2	ND	32.2	10.3	1.3	ND
	ChCl/LA	3.7	22.8	7.7	ND	47.5	14.5	7.8	ND
	ChCl/urea	6.5	<LOQ	7.7	ND	39.5	12.5	6.0	<LOQ
	Bet/Gly	7.6	<LOQ	6.0	ND	34.9	10.9	1.3	ND
	Bet/LA	5.7	<LOQ	7.4	ND	41.7	11.2	5.7	ND
	ChCl/AA	12.3	<LOQ	12.5	ND	58.3	18.2	10.0	<LOQ
	Bet/AA	0.1	23.4	11.3	ND	56.0	15.8	12.2	<LOQ
Fresh Petit Verdot	Ethanol/water 50%	6.3	23.1	16.3	ND	77.2	27.8	14.2	68.3
	Ethanol/water 70%	8.9	28.1	13.6	ND	68.9	25.5	13.1	85.7
	ChCl/Gly	7.4	<LOQ	<LOQ	ND	37.3	9.9	5.5	ND
	ChCl/LA	9.4	30.9	7.7	ND	54.2	14.1	11.5	ND
	ChCl/urea	10.3	<LOQ	10.2	ND	48.5	17.4	10.1	ND
	Bet/Gly	7.5	27.0	8.7	ND	50.1	16.9	13.4	ND
	Bet/LA	6.3	24.6	9.7	ND	53.5	18.3	17.5	<LOQ
	ChCl/AA	13.8	41.4	6.8	ND	68.3	26.5	20.4	15.5
	Bet/AA	0.1	49.5	10.5	ND	64.6	26.5	26.1	66.9
Dried Cabernet Sauvignon	Ethanol/water 50%	47.0	38.8	15.5	ND	54.9	28.8	14.3	174.2
	Ethanol/water 70%	53.4	46.2	20.0	ND	64.2	36.0	20.2	198.6
	ChCl/Gly	34.6	30.4	12.6	ND	35.7	16.2	6.8	<LOQ
	ChCl/LA	38.2	52.8	13.0	ND	51.8	22.2	12.7	91.9
	ChCl/urea	48.8	48.7	18.7	ND	70.4	28.1	14.8	88.9
	Bet/Gly	38.0	31.9	8.1	ND	27.5	11.0	7.9	<LOQ
	Bet/LA	61.1	61.1	16.7	ND	78.0	32.2	21.9	204.5
	ChCl/AA	50.2	32.3	12.3	ND	40.4	29.8	18.3	131.5
	Bet/AA	79.1	73.2	10.6	5.2	48.3	26.8	12.9	118.9
Dried Petit Verdot	Ethanol/water 50%	65.9	46.4	32.1	ND	84.6	42.9	21.7	265.8
	Ethanol/water 70%	67.4	27.0	32.6	ND	84.0	44.9	23.5	492.5
	ChCl/Gly	44.6	34.2	15.9	ND	40.0	17.1	8.1	74.6
	ChCl/LA	46.7	52.4	18.9	ND	69.8	31.8	19.0	126.5
	ChCl/urea	52.9	51.8	22.4	ND	62.6	27.3	14.8	80.1
	Bet/Gly	54.0	27.6	15.8	ND	50.8	24.1	15.9	ND
	Bet/LA	67.3	57.2	20.1	ND	80.2	37.7	24.3	170.8
	ChCl/AA	49.3	52.2	11.5	ND	44.3	39.5	18.4	156.5
	Bet/AA	93.0	70.7	15.0	6.4	66.6	35.3	17.1	155.4

ND: not detected; LOQ: limit of quantitation; all the DESs included +40% water.

**Table 3:** Antioxidant activity (results are reported as gallic acid equivalents).

GRAPE POMACE	SOLVENT	μM GAE	GRAPE POMACE	SOLVENT	μM GAE
Fresh Cabernet Sauvignon	Ethanol/water 50%	ND	Dry Cabernet Sauvignon	Ethanol/water 50%	5080 ± 1200
	Ethanol/water 70%	ND		Ethanol/water 70%	5980 ± 840
	ChCl/Gly	166 ± 23		ChCl/Gly	6960 ± 1450
	ChCl/LA	1710 ± 150		ChCl/LA	8170 ± 1270
	ChCl/urea	1910 ± 290		ChCl/urea	12,550 ± 2360
	Bet/Gly	3530 ± 370		Bet/Gly	8960 ± 1350
	Bet/LA	5060 ± 440		Bet/LA	9800 ± 1160
	ChCl/AA	ND		ChCl/AA	ND
	Bet/AA	ND		Bet/AA	ND
Fresh Petit Verdot	Ethanol/water 50%	1530 ± 80	Dry Petit Verdot	Ethanol/water 50%	5880 ± 280
	Ethanol/water 70%	990 ± 70		Ethanol/water 70%	4740 ± 210
	ChCl/Gly	5340 ± 270		ChCl/Gly	3920 ± 180
	ChCl/LA	6630 ± 230		ChCl/LA	4020 ± 160
	ChCl/urea	6970 ± 180		ChCl/urea	15,000 ± 1030
	Bet/Gly	1850 ± 150		Bet/Gly	2350 ± 93
	Bet/LA	720 ± 85		Bet/LA	3390 ± 280
	ChCl/AA	ND		ChCl/AA	ND
	Bet/AA	ND		Bet/AA	ND

ND: not determined; all the DESs included + 40% water.

**Table 4:** Antibacterial activity (MIC and MBC in % *v*/*v*) of solvents used in this study.

	*E. faecalis* 29212		*S. aureus* 29213		*S. aureus* 43300		*E. coli* 25922		*K. pneumoniae* 13883		*P. aeruginosa* 27853	
^a^ Solvent	MIC	MBC	MIC	MBC	MIC	MBC	MIC	MBC	MIC	MBC	MIC	MBC
70% EtOH	12.5	25.0	12.5	25	25	50	25	50	25	50	25	50
ChCl/AA	1.6	3.1	1.6	3.1	3.1	6.3	3.1	6.3	3.1	6.3	6.3	12.5
Bet/LA	0.4	0.8	0.4	0.8	0.4	0.8	0.4	0.8	0.4	0.8	0.8	1.6
ChCl/Urea	6.3	12.5	6.3	12.5	12.5	25	12.5	25	12.5	25	12.5	25

^a^ ChCl/AA: choline chloride–ascorbic acid + 40% water; Bet/LA: betaine–lactic acid + 40% water; ChCl/Urea: choline chloride–urea + 40% water.

**Table 5:** Antibacterial activity (MIC and MBC in % *v*/*v*) of grape pomace extracts in DESs or 70% EtOH and levofloxacin (µg/mL).

	*E. faecalis* 29212		*S. aureus* 29213		*S. aureus* 43300		*E. coli* 25922		*K. pneumoniae* 13883		*P. aeruginosa* 27853	
Extract (^a^ Solvent)	MIC	MBC	MIC	MBC	MIC	MBC	MIC	MBC	MIC	MBC	MIC	MBC
119 (70% EtOH)	0.8	1.6	0.4	0.8	0.4	0.8	1.6	3.1	3.1	6.3	3.1	6.3
122 (ChCl/AA)	0.8	1.6	0.4	0.8	0.4	0.8	0.8	1.6	1.6	3.1	1.6	3.1
140 (Bet/LA)	0.2	0.4	0.1	0.2	0.1	0.2	0.2	0.4	0.2	0.4	0.4	0.8
152 (ChCl/Urea)	0.8	1.6	0.4	0.8	0.4	0.8	1.6	3.1	3.1	6.3	3.1	6.3
levofloxacin	2.0	-	0.5	-	1.0	-	0.1	-	8.0	-	4.0	-

^a^ ChCl/AA: choline chloride–ascorbic acid + 40% water; Bet/LA: betaine–lactic acid + 40% water; ChCl/Urea: choline chloride–urea + 40% water.

**Table 6:** Deep eutectic solvents (DESs)’ composition.

HBA	HBD	Molar Ratio	Water Content (%)	Reference
ChCl	AA	2:1	40	[31]
Bet	AA	2:1	40	[31]
Bet	Gly	1:4	40	[32]
Bet	LA	1:4	40	[32]
ChCl	Gly	1:2	40	[33]
ChCl	LA	1:2	40	[34]
ChCl	Urea	1:2	40	[21]

**Table 7:** LOD and LOQ of the phenolic compounds.

Phenolic Compounds	LOD (ng/g)	LOQ (ng/g)
Gallic acid	0.18	0.37
Catechin	10.1	20.3
Syringic acid	0.89	1.78
Rutin and quercetin glucosides	2.58	5.16
Quercetin	30.9	61.8
Kaempferol 3-galactoside	19.3	38.6
Apigenin	7.56	15.1
Apigenin 3-glucuronide	1.03	2.07
Resveratrol	0.06	0.12
Ferulic acid	0.19	0.39
Chlorogenic acid	1.39	2.78
Caffeic acid	0.67	1.33
Coumaric acid	0.32	0.64

LOD = limit of detection; LOQ = limit of quantification.

## Data Availability

All data supporting the findings of this study are available within the paper.
